# Knowledge of nosocomial infections, standard precautions, and source of information among physiotherapy undergraduates in Sri Lanka; an observational study

**DOI:** 10.1186/s13756-023-01248-6

**Published:** 2023-05-09

**Authors:** Sahan Rubasinghe, Kokila Priyadarshani, Pramodha Wijesundara, Singappulige Ramyamala, Krishantha Lakmal, Anuradhi Bandara, Renuka Dasanayaka

**Affiliations:** Department of Physiotherapy, Faculty of Allied Health Sciences, University of Peradeniya, Peradeniya, 20400 Sri Lanka

**Keywords:** Knowledge, Nosocomial infections, Physiotherapy undergraduates, Sources of information, Standard precautions

## Abstract

**Background:**

Physiotherapists and physiotherapy undergraduates have direct contact with patients which make them transmitters of infections if they do not follow standard precautions. Hence, the purpose of this study was to assess the knowledge of nosocomial infections, standard precautions, and source of information among physiotherapy undergraduates in Sri Lanka.

**Methods:**

An observational Google based survey study was conducted among 294 physiotherapy undergraduates, of which there were 103 in University of Peradeniya, 103 in University of Colombo, and 88 in General Sir John Kotelawala Defence University. The Infection Control Standardized Questionnaire comprising three domains: knowledge of nosocomial infections, standard precautions and hand hygiene was used for data collection along with a self-constructed data sheet for socio-demographic information and source of information.

**Results:**

Participants achieved mean knowledge of 67.1 ± 16.8, 84.4 ± 14.7 and 66.4 ± 15.4 for nosocomial infections, standard precautions, and hand hygiene respectively. Of the total sample, 225 (76.5%) achieved adequate level of total knowledge. Eighty-three of them (28.3%) equally mentioned, formal teaching at faculty and informal sources as the most important source of knowledge. There was no significant impact of university and the duration of clinical exposure on knowledge of nosocomial infections, standard precautions, hand hygiene and total knowledge. The study year has a significant impact on standard precautions (*P* = 0.004) and total knowledge (*P* = 0.035) and final years had highest knowledge compared to the other study years.

**Conclusion:**

Knowledge of nosocomial infections and infection control measures were satisfactory among the physiotherapy undergraduates in Sri Lanka. Further developments of formal sources of information about nosocomial infections are recommended.

## Background

Infections that are acquired during hospital care after 48 h of admission, which are not presented or incubated at the time of admission and infections that occur within 30 days of discharge after inpatient care are known as nosocomial infection (NI) [1, 2]. NIs develop through exposure to microorganisms such as bacteria, virus, and fungi in healthcare settings [2]. Micro-organisms are transmitted via various routes of transmission such as contact (direct and indirect), droplet and airborne routes [3]. Lower respiratory tract infection (LRTI), urinary tract infections (UTI), surgical site infections and blood stream infections can occur as a result [4]. In addition, COVID-19 has alarmed the healthcare community of the danger and harm of nosocomial infections. Nosocomial transmission of COVID-19 has been reported in numerous healthcare facilities on a global scale.

Transmission of infections and its severity can be prevented with good hygienic practices, and proper knowledge of the use of gloves, protective clothing and alcohol based hand rubs and invasive procedures [5]. In addition, transmission of infections can be prevented by following infection control guidelines, adhering to the standard precautions, continuing education and arranging updated training facilities for health care professionals [1, 6].

In Sri Lanka, multidisciplinary teams work together to take care of patients and these hospital environments provide a favorable transmission pathway for nosocomial infections, due to poor practices of infection control among health care workers as well as overcrowding of patients in most clinical settings [5]. Physiotherapists and physiotherapy students have close contact with patients throughout the examination and treatment process, similar to all other healthcare professionals [7]. If they have sufficient knowledge of standard precautions, they can prevent transmission between patients [7].

Although there are several studies that have assessed knowledge of NIs and standard precautions among other health care professionals and health care students worldwide and in Sri Lanka [1, 6–11], there are only a few studies carried out among physiotherapy professionals and students in this field [7]. Further, these studies encourage researchers to conduct more studies in this field to measure knowledge and practice and to identify the gaps to address them [7].

In addition, source of information is a major factor that impacts knowledge about prevention of NIs and standard precautions [9]. Different studies found that students gained knowledge through different resources and some of these were informal resources. There is a risk of reliability and validity of the information that students acquire when they gain knowledge via informal sources [9]. Hence, this study aimed to assess the knowledge of nosocomial infections and standard precautions and source of information among physiotherapy undergraduates.

## Methods

The aim of this study was to assess the knowledge of nosocomial infections, standard precautions and sources of information among physiotherapy undergraduates in Sri Lanka and to find out how the university, study year, gender and duration of clinical exposure had an influence on the knowledge of nosocomial infections and standard precautions. University of Peradeniya, University of Colombo and General Sir John Kotelawala Defence University are University Grant Commission recognized institutes which produce graduated physiotherapy professionals in Sri Lanka. The 1st year, 2nd year, 3rd year and 4th year Bachelor of Science Physiotherapy undergraduates in all the three universities were taken as the study population for this observational study.

Infection control standardized questionnaire (ICSQ) which had been prepared according to international guidelines on standard isolation precautions and hand hygiene was used as the data collection tool to measure the knowledge of nosocomial infections and standard precautions [12]. The questionnaire has three main domains with 25 close-ended questions; knowledge about nosocomial infection (5 questions), standard precautions (12 questions) and hand hygiene (8 questions). The answer to each question was coded & scored as a correct answer (2), and incorrect answer (0). The maximum possible score achievable was 50. Participants who scored 35(70%) or more were rated as having satisfactory knowledge on the subject [12]. Data on demographic information, clinical exposure, and the source of information were collected by a Google form-based survey along with the ICSQ.

All statistical analyses were conducted with Statistical Package for Social Sciences (SPSS) statistical software version 22. Descriptive statistics was used to summarize the data and to find the distribution of data. Knowledge of nosocomial infection and standard precautions among the study years and universities was analyzed using Analysis of Variance (ANOVA). Pearson correlation coefficient test was used to analyze the association among duration of clinical exposure, knowledge of nosocomial infections and standard precautions.

## Results

**Fig. 1:**
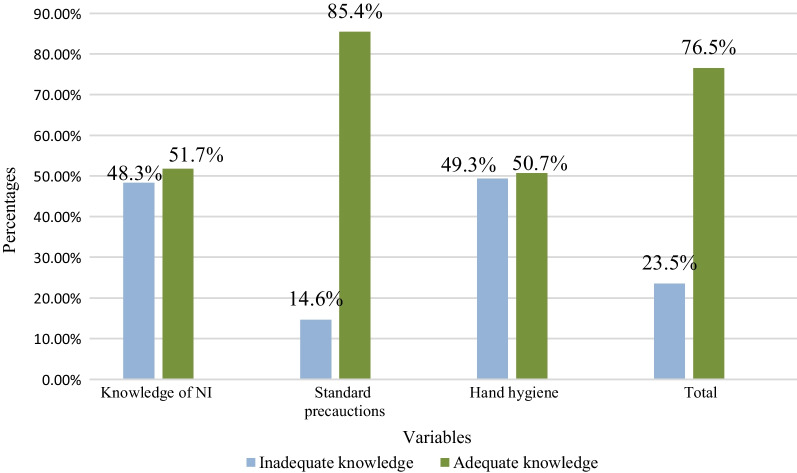
Classification of knowledge of nosocomial infection and standard precautions

Two hundred and ninety four students (103 students in Peradeniya, 103 students in Colombo and 88 students in General Sir John Kotelawala Defence University) were included in the study. Participants had higher mean knowledge (84.4 ± 14.7) of standard precautions than NIs (67.1 ± 16.8) and hand hygiene (66.4 ± 15.4). The total mean knowledge was 75.2 ± 10.0 and a score above 70% was considered as adequate knowledge and below 70% as inadequate. Two hundred and twenty-five (76.5%) participants achieved adequate level of total knowledge. Two hundred and fifty-one (85.4%) participants had adequate knowledge on standard precautions which was higher than the number of participants with knowledge on NIs and hand hygiene (Fig. 1).

**Fig. 2:**
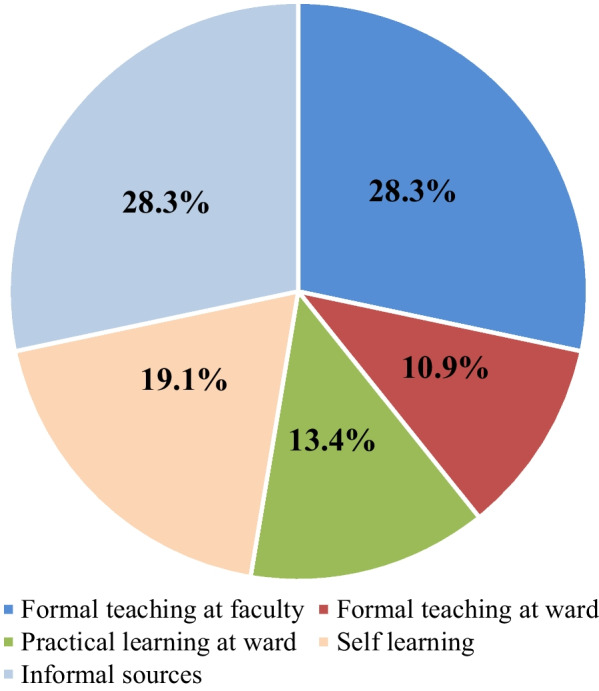
Percentage of source of information

Most students reported that the most important source of knowledge for each area was formal teaching at the university and informal sources (28.3%), and few reported that the most important source was formal teaching at hospital wards (10.9%). The alternative sources of information were self- learning (19.1%) and practical learning in the ward (13.4%) (Fig. 2). There was no statistically significant difference in the knowledge of NI, standard precautions, hand hygiene and total knowledge based on the university where the participants were studying. The study year had statistically significant impact on knowledge of standard precautions (*p* = 0.004) and total knowledge (*p* = 0.035) with final year students demonstrating higher level of knowledge compared to other study years**.** There was no statistically significant impact on knowledge of NI and knowledge of hand hygiene (Table 1). There was a significant difference in knowledge of standard precautions (*p* = 0.004) with 4th year students demonstrating more knowledge compared to 2nd year students. There was no significant relationship between the duration of clinical exposure and knowledge of nosocomial infections, standard precautions, hand hygiene and total knowledge. Females demonstrated higher level of knowledge about hand hygiene and there was a significant difference of total knowledge between males and females. Informal sources significantly contributed to an increase in the total knowledge. Total knowledge of 2nd year students was less than 1st year students.

**Table 1:** Comparison of the knowledge of NIs, standard precautions and hygiene with different study years

Testing variables	Grouping variable; study year (mean ± SD)				ANOVA test (*p* value)
	1st year (n = 73)	2nd year (n = 92)	3rd year (n = 74)	4th year (n = 55)	
Knowledge of NIs	67.4 ± 14.7	67.8 ± 17.3	65.7 ± 17.7	67.6 ± 17.8	0.856
Knowledge of standard precautions	84.9 ± 14.3	80.7 ± 16.9	84.6 ± 15.3	89.7 ± 6.8	0.004*
Knowledge of hand hygiene	66.8 ± 13.9	64.8 ± 17.3	67.7 ± 15.5	66.8 ± 14.1	0.657
Total knowledge	75.6 ± 9.5	73.0 ± 11.2	75.4 ± 10.2	77.9 ± 7.5	0.035*

NIs: Nosocomial infections, n: sample size, SD: standard deviation

^*^Variable with significant difference, checked at 5% significant level and 95% confidence interval

## Discussion

In this study, the participants had an mean total knowledge of 75.2 ± 10.0 with higher knowledge (84.4 ± 14.7) of standard precautions confirmed by other studies with similar findings [6, 9, 12]. The higher level of knowledge of students might be due to formal teaching at university and clinical setting and increased awareness regarding standard precautions due to COVID-19 pandemic. Studies conducted among physiotherapy students in different countries with different educational facilities and backgrounds showed similar level of knowledge about infection control [6, 9, 12]. Another study conducted among health care students who engaged in clinical training found highest scores in knowledge about standard precautions followed by hand hygiene and NIs which is similar to our study findings [12]. More than 50% of the participants (n_i_ = 152, 252, 149, 225) had scored adequate knowledge in each domain; NIs, standard precautions, hand hygiene and total. Seventy six point five percent of participants achieved adequate level of knowledge and similar observations can be seen in previous studies [6, 9, 12].

The most important source of knowledge for each area was formal teaching at a university and informal sources like social media (28.3%). Few participants reported that the most important source was formal teaching in the ward (10.9%) which is relatively similar to previous studies [9, 12]. In addition, universities were closed for around one year due to the COVID-19 pandemic where students were not directed to go for clinical training in the hospital wards in the proper sequence. Hence, the percentage of students who gathered their knowledge through formal teaching at wards had drastically reduced. All these studies found that knowledge gathered via formal sources are comparatively low even in the countries with well-established curricula and facilities [6]. In contrast, students are more prone to search knowledge via informal sources and that may be the reason why 20–30% did not have adequate knowledge. Further improvements of formal training sources seem essential.

There was no statistically significant difference in knowledge of NIs, standard precautions, hand hygiene, and the total knowledge among the universities. This could be because undergraduates in all three universities get their clinical exposure through the same government setting and they all follow curricula with similar content. In contrast, the total knowledge had increased due to increased awareness on infection control during COVID-19 pandemic. However, another study found there is a statistically significant relationship between knowledge level and teaching program [13]. There was a significant impact of the study year on knowledge of standard precautions and total knowledge and no significant impact of the study year on knowledge of NI and hand hygiene. The study found that the final year students had higher level of knowledge compared to other study years. These findings might be a result of more clinical exposure among final year students compared to the other study years and the application of their theoretical knowledge into practice during their clinical rotation. Further, results have indicated that the total knowledge of 2nd year students was less than the 1st year students. Most of the curricula have infection control teaching sessions during the 1st year as well as the 3rd year. Second years students had less knowledge compared to 1st and 3rd years and final years had highest as they practically apply the knowledge in clinical settings. Uneven distribution of knowledge can be the result of the above mentioned facts. As most of the studies compared the knowledge among undergraduates with different disciplines, no studies had compared how knowledge varied between the study years.

There was no statistically significant relationship between duration of clinical exposure and knowledge of NIs, standard precautions, hand hygiene, and total knowledge. In contrast, another study showed statistically significant relationship between knowledge and clinical training program based on 565 healthcare students [13]. Clinical exposure of the 3rd and 4th year students was disturbed with COVID-19 pandemic and political insecurity within the country. This could result in an insignificant correlation between knowledge and duration of clinical exposure.

Females had a higher level of knowledge on hand hygiene and there was a significant difference of total knowledge between males and females. Two thirds of the study participants were females and the big difference between male: female proportion could have impacted significantly on the conclusion. Moreover, females were more compliant to hygienic practices and the study outcomes were influenced by such factors. A similar study among healthcare students (394 females and 171 males) conducted in Cukurova University found that women had a statistically significant higher level of knowledge compared to men [13]. In contrast, another study conducted amongst health science students (122 females and 40 males) in Namibia found that there was no significant relationship between gender and knowledge [11].

Though the study have presented how different factors impact knowledge of NIs, standard precautions and hand hygiene, the COVID-19 pandemic can be considered as a major drawback where the study could not find exact significant factors.

## Conclusion

The study concluded that knowledge of NIs, and infection control measures were satisfactory among most of the physiotherapy undergraduates in Sri Lankan government universities. Nevertheless, the knowledge on NIs, and hand hygiene was lower compared to the knowledge on standard precautions. The study showed the need for further development of formal sources of information on NIs. In order to enhance knowledge on the prevention of NIs among physiotherapy undergraduates, it is suggested to introduce infection control as a module at the university level at the earliest opportunity. Further, instructions should be given to adhere to infection control guidelines prior to commencing inwards clinical training at hospitals. In addition, continuous education programs, seminars, or workshops should be conducted to update the knowledge on regular basis.

## Data Availability

The datasets used and/or analysed during the current study are available from the corresponding author on reasonable request.

## Abbreviations

ANOVA: Analysis of variance
COVID-19: Coronavirus disease 2019
ICSQ: Infection control standard questionnaire
LRTI: Lower respiratory tract infection
n: Number of participants
NI: Nosocomial infection
SD: Standard deviation
SPSS: Statistical package for social sciences
UTI: Urinary tract infection
